# Oral lesions and disorders and their prevalence arising from the use of illicit drugs in a prison population

**DOI:** 10.2340/aos.v84.42721

**Published:** 2025-01-24

**Authors:** Marta Relvas, Luciana Rocha, Paulo Rompante, Filomena Salazar, Luís Monteiro, Ana Sofia Vinhas, Maria Gonçalves

**Affiliations:** a Oral Pathology and Rehabilitation Research Unit (UNIPRO), University Institute of Health Sciences (IUCS-CESPU), 4585-116 Gandra, Portugal; b Associate Laboratory i4HB—Institute for Health and Bioeconomy, University Institute of Health Sciences—CESPU, Gandra 4585-116, Portugal; c UCIBIO—Applied Molecular Biosciences Unit, Translational Toxicology Research Laboratory, University Institute of Health Sciences (1H-TOXRUN, IUCS—CESPU), Gandra, Portugal

**Keywords:** Illicit drugs, prison population, oral disorders, oral lesions

## Abstract

**Background:**

The dependence on illicit drugs has been proven to be harmful to the oral cavity and may lead to a series of abnormal manifestations. The main objective of this study was to observe the effects caused by the consumption of illicit drugs in the oral cavity, in a prison population in the North of Portugal.

**Methods:**

A cross-sectional observational study was conducted involving 91 male inmates aged 25–75 years (mean age 41.14 ± 8.98), from Paços de Ferreira Prison. The participants were subjected to a medical interview and a clinical examination. Descriptive statistics and Chi-square test were used to assess the association between the qualitative variables. The level of statistical significance used was α ≤ 0.05.

**Results:**

The consumption of illicit drugs proved to be a common practice (86.8%), where cannabis, heroin and cocaine were the most prevalent (29.1%). Thirty-one inmates were attending the methadone rehabilitation program (34.1%). Most of the patients consuming illicit substances were also smokers (93.7%) (*p* < 0.001). Of the 91 participants, 82 (90.1%) presented oral manifestations, with caries lesions being the most prevalent (61.0%) and oral mucosa lesions the least observed (3.7%). Heavy drug consumption was related to rampant caries lesion (*p* = 0.011) and chewing difficulty (*p* = 0.024) when compared with cannabis consumption.

**Conclusion:**

The main oral manifestations found, associated with the consumption of illicit drugs, were caries lesions, tooth loss and sensitivity, periodontal diseases, temporomandibular disorders, xerostomia, and bruxism.

## Introduction

The consumption of illicit drugs by the prison population is one of the major health problems, both for its frequency and severity [1–3]. However, there is controversy about its prevalence, which shows a scarcity of data on the consumption of those substances during imprisonment [4]. This variation may be due to not only the research methodology, but also the greater difficulty in acquiring substances, or the inmate’s inherent fear of being detected and penalized [1, 5].

The use of illicit drugs usually starts during adolescence and young adulthood, with low educational level as an increased risk factor [6].

Drug use has been shown to be harmful to many different parts of the human body, including the oral cavity, giving rise to a series of abnormal manifestations that can directly or indirectly affect the parts of the mouth, embracing the mucous membranes, teeth, and periodontal tissues and, when left untreated over time, typically become more severe, more painful, more difficult to treat and more costly, and can severely affect masticatory function, esthetics, and general well-being [6–8].

Although illicit drugs vary in terms of composition, route of administration and purpose, there seems to be a common profile of manifestations, with some differences. In cannabis users, there are caries lesions, periodontal disease, xerostomia and candidiasis [9]. In cocaine users, we can observe perforation of the palate and nasal septum, periodontal disease, high rate of caries and tooth loss, bruxism, temporomandibular disorders (TMD), paresthesia and decreased taste sensitivity [8, 10, 11]. In those on heroin, in addition to periodontal disease, xerostomia and tooth loss, a pathognomonic sign is cervical caries lesions [10]. Meth mouth is the term used to describe oral lesions in methamphetamine users [3, 8, 10]. In addition to bruxism, TMD, tooth attrition, xerostomia and periodontal disease, caries lesions and tooth erosion also appear to be common in ecstasy users [10].

Oral mucosal lesions, some of them pre-cancerous, can also be found in this range of consumers [6, 11].

There are also two common behaviors among the inmate population that have implications for oral health: smoking and alcohol consumption. Several studies indicate that excessive alcohol consumption increases the prevalence and severity of periodontal disease and increases the risk of oral cancer [8, 11]. It has been observed that this population does not have oral hygiene habits, which also contributes negatively to the appearance or worsening of these manifestations [12].

In Portugal, the high proportion of inmates with a history of illicit drug use have led to the emergence of a growing need to implement drug treatment programs in prison [13]. Thus, regarding the intervention programs with the drug-addicted inmate population, the Director-General of Reinsertion and Prison Services (DGRPS) has an abstinence-oriented program where methadone is used. This substance is given to patients as a synthetic opioid with prolonged action, but which by itself, can trigger oral repercussions such as xerostomia, caries lesions and periodontal disease [2, 9].

Under ideal conditions, prisons should provide an opportunity for addicted inmates to receive appropriate treatment, facilitating their subsequent social reintegration after serving their sentence. Oral health is extremely important for individual well-being and enhances a healthier social interaction and a more peaceful reintegration [1].

This study has as its main objective the observation of the effects caused by illicit drugs in the oral cavity, due to its consumption, in a prison population in the North of Portugal, having also as secondary objective the evaluation of potential aggravating factors such as smoking and alcohol consumption.

## Methods

### Study design and ethical approval

This study was conducted among the prisoners in Prison of Paços de Ferreira (EPPF) during the period from October 2022 to December 2023.

The study was approved by the ethics commission of the University Institute of Health Sciences, with reference number CE/IUCS/CESPU-20/21, and by the General Directorate of Reintegration and Prison Services. The study adhered to the ethical principles of the Declaration of Helsinki.

### Study population and data collection

Participants were informed through oral and written explanations about the purpose and procedures of the study. Those who agreed to participate in the study were asked to sign an informed consent form and to complete a questionnaire before the clinical examination. Inclusion criteria were those inmates present at the EPPF during the data collection period and those who were aged 18 years and older. Participants would also have to answer affirmatively to one of the following criteria: previous consumption of alcohol, tobacco and/or illicit drugs and the detection of the presence of oral manifestations. Exclusion criteria were those individuals under disciplinary isolation. The subjects who did not give their consent were excluded.

The sample was selected according to a non-probability convenience sampling method from the inmate population of the EPPF. The study population in this cross-sectional observational study comprised 91 male prisoners. From an initial sample of 107 inmates, four did not agree to participate in the study, and from the remaining 103, 12 did not meet the established inclusion criteria. Details of the nature and purpose of the study were described verbally, and in writing, using the consent form. Written informed consent was obtained from all participants before the oral examination and administering the questionnaire.

The sample was subjected to a clinical examination and a questionnaire was used to collect data. Data collection was done by a dentist (M.R.) under the supervision of a specialist in oral pathology (L.M.). The following information was collected: sociodemographic data; level of education (1st cycle [4 years of education], 2nd cycle [6 years of education], 3rd cycle [9 years of education], secondary education [12 years of education], higher education [University education]); smoking habits; alcohol consumption habits; consumption of illicit drugs; frequency of rehabilitation program; oral signs and symptoms.

The variables collected through clinical examination included the following: number of decayed teeth; number of teeth with mobility; pocket depth (PPD), measured as the distance from the gingival free-margin from the bottom of the pocket; gingival recession (REC) as the distance from the enamel-cement junction (CEJ) to the free gingival margin; and clinical attachment loss (CAL). Periodontal parameters were recorded by means of a periodontal probe with 1-mm markings (PCP-UNC 15, Hu-Friedy, Chicago, IL, USA) at six sites per tooth. Furthermore, visible clinical manifestations most commonly related to the consumption of illicit substances (cannabis, cocaine, heroin, methamphetamines, ecstasy and others); precancerous lesions; inflammation (enanthema) and lesions of the oral mucosa (ulcers); oral candidiasis; caries lesions; bruxism; perforation of the palate and nasal septum; tongue hyperpigmentation and rampant caries lesions were also recorded. It is also worth mentioning the presence of other manifestations with oral repercussions, but which may or may not be detectable with the naked eye, such as: xerostomia, periodontal diseases, TMD, lingual and labial paresthesia, decreased taste sensitivity, dental hypersensitivity, and facial tremors.

Periodontal health, gingivitis and periodontitis were defined according to the new consensus of the American Academy of Periodontology / European Federation of Periodontology (AAP/EFP) [14]. Periodontal health was defined as follows: when the total percentage of bleeding in probe was <10% and probing depth ≤ 3 mm. Gingivitis was indicated when the total percentage of bleeding in probe was ≥10% and PPD < 4 mm. Periodontitis was considered to be present when interproximal CAL was detectable in two or more interproximal sites not adjacent or when there was an interproximal CAL of 2 mm or more, non-vestibular or lingual/palatal, for ≥ 2 teeth. Periapical radiographs were taken to measure interproximal bone loss.

### Statistical analysis

The data were analyzed using IBM^®^ SPSS statistics software (Statistical Program for Social Sciences) version 28.0 for Windows. Descriptive statistics were expressed as mean. standard deviation (SD) for quantitative variables and as frequencies and percentages for qualitative variables. To verify the existence or not of a relationship between the type of substances consumed and the most prevalent oral manifestations, we grouped the substances into two: cannabis and hard drugs. The Chi-square test was used to assess the association between the qualitative variables such as age groups, alcohol consumption habits, smoking, consumption of illicit substances, frequency of the rehabilitation program, oral hygiene habits, according to the most prevalent oral manifestations. The level of statistical significance used was α ≤ 0.05.

## Results

### Demographic and socioeconomic data

Our sample was composed of 91 inmates aged between 25 and 75 years (mean = 41.14; SD = 8.98). Most (40.7%) of the participants were aged between 36 and 44 years (Table 1). As regards education, 26 (28.6%) participants had attended the 3rd cycle, 25 (27.5%) had attended the 2nd cycle, and 22 (24.3%) had attended the primary school. Only three (3.3%) of the participants had completed higher education and 15 (16.5%) had completed secondary education (Table 1).

**Table 1:** Demographic characteristics of the study population.

Characteristics	*n*	%
**Age group (years)**		
20–35	28	30.8
36–44	37	40.7
>44	26	28.6
**Education level**		
1^st^ Cycle^1^	22	24.2
2^nd^ Cycle^2^	25	27.5
3^rd^ Cycle^3^	26	28.6
Secondary Education^4^	15	16.5
Higher Education^5^	3	3.3
**Use of tobacco**		
Yes	81	89.0
No	10	11.0
**Cigarretes used per day**		
0–9	24	26.4
10–20	40	44.0
>20	17	18.7
**Drinking alcohol**		
No	83	91.2
Rarely	1	1.1
Sometimes	5	5.5
Once a week	2	2.2
**Consumption of illicit substances**		
Yes	79	86.8
No	12	13.2
**Years of consumption**		
< 10	36	39.6
10–20	30	33.0
> 20	13	14.3
**Rehabilitation program**		
Yes (Methadone)	31	34.1
No	48	52.7
**Frequency of tooth brushing**		
Never	14	15.4
Once a day	16	17.6
Twice a day	33	36.3
Thrice a day	24	26.4
3 or more times a day	2	2.2
2 or more times a week	2	2.2
**Auxiliary means other than using brush and toothpaste**		
Yes	42	40.8
No	49	47.6
**What auxiliary means**		
Mouthwash	29	28.2
Dental floss	7	6.8
Mouthwash and Dental floss	6	5.9

%: percent; *n*: number.

1 4 years of education;

2 6 years of education;

3 9 years of education;

4 12 years of education;

5 University education.

### Habits: Smoking, consumption of alcohol and illicit substances and oral health

**Figure 1:**
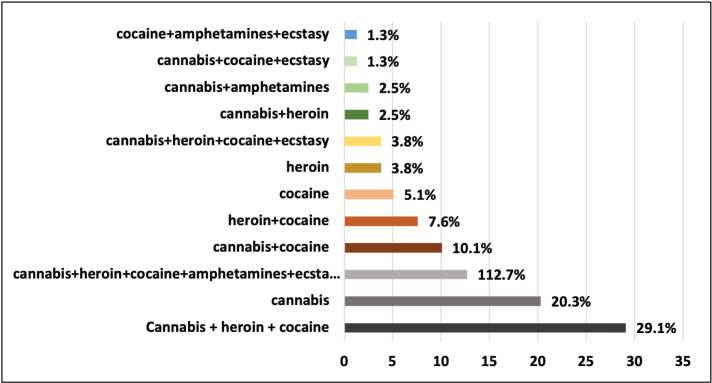
Type of illicit substances consumed.

Most of the participants (89.0%) were smokers. Of these, 48 (52.7%) had been smoking for less than 25 years, and 40 (44.0%) smoked between 11 and 20 cigarettes a day. As for the consumption of alcoholic drinks, 83 inmates (91.2%) reported not consuming them, and only two participants (2.2%) reported weekly consumption. The consumption of illicit substances was found to be a common practice among the participants, with 79 (86.8%) inmates reporting that they had been already consuming one or more types of illicit substances, of which 36 (39.6%) of them had started using them 10 years ago or less. Regarding the type of illicit substances most consumed (Figure 1), the combination of ‘cannabis, heroin and cocaine’ predominate with 29.1%. Cannabis alone accounts for 20.3% of those drugs consumed. The main illicit drugs considered in this study (cannabis, heroin, cocaine, amphetamines, and ecstasy) accounted for 12.7% of the drugs which are consumed. Cannabis and cocaine together were consumed by 10.1% of the participants. At the other extreme, with just 1.3%, there was consumption of ‘cannabis, cocaine and ecstasy’ as well as ‘cocaine, amphetamines and ecstasy’.

Of the 79 users of illicit drugs, 31 (34.1%) were attending an abstinence-oriented rehabilitation program (methadone program) (Table 1).

From the analysis of Table 1, regarding the oral hygiene habits, 77 (84.6%) of the participants mentioned brushing their teeth, and of these, 33 (36.3%) said they do it twice a day, and 42 (40.8%) said they use mouthwash and/or dental floss as an auxiliary means of brushing, in addition to using brush and toothpaste.

### Prevalence of oral manifestations

**Figure 2:**
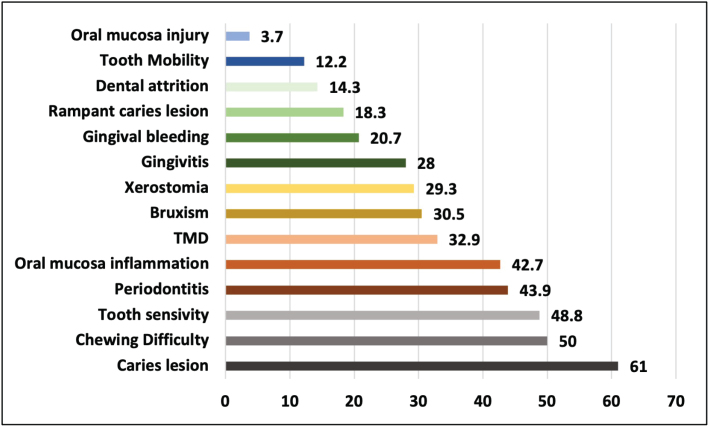
Prevalence of oral lesions and disorders among the study sample

Of the 91 participants, 82 (90.1%) had oral manifestations, the most prevalent being caries lesions (61.0%). Difficulty chewing, essentially due to the lack of dental pieces, and tooth sensitivity were also prominent with 50.0% and 48.8%, respectively. The prevalence of periodontal diseases was also notable, with 36 inmates (43.9%) showing periodontitis and 23 (28.0%) gingivitis, TMD (32.9%) and bruxism (30.5%), which were also reported with some frequency, should also be noted, as well as xerostomia (29.3%). Dental attrition was also observed in 13 (14.3%) respondents (Figure 2).

### Relationship between consumption of illicit substances and smoking, alcohol consumption and age

Of the 79 participants who reported consuming illicit substances, 74 (93.7%) were smokers, and this relationship was statistically significant (χ^2^(1) = 13.3 *p* < 0.001). Regarding the consumption of alcoholic beverages, only eight participants reported consuming them, of which seven (87.5%) consumed illicit substances, although this relationship was not statistically significant (Table 2).

**Table 2:** Substance use versus tobacco, alcohol, and age.

		Consumption of illicit substances		Consumption of illicit substances (years)			Total		
		No	Yes	≤10	11–20	>20	*n* (%)	χ^2^	*p*
		*n* (%)	*n* (%)	*n* (%)	*n* (%)	*n* (%)			
**Smoking**	**No**	5 (41.7)	5 (6.3)				10 (11.0)	13.3	<0.001
	**Yes**	7 (58.3)	74 (93.7)				81 (89.0)		
**Alcohol consumption**	**No**	11 (91.7)	72 (91.1)				83 (91.2)	0.004	0.952
	**Yes**	1 (8.3)	7 (8.9)				8 (8.8)		
**Age group (years)**	**25–35**			14 (51.9)	13 (48.1)	0 (0.0)	27 (100.0)	8.26	0.015
	**36–44**			13 (41.9)	10 (32.3)	8 (25.8)	31 (100.0)		
	**>44**			9 (42.9)	7 (33.3)	5 (23.8)	21 (100.0)		
	**Total**			36	30	13	79		

*n*: frequency; %: percentage; χ^2^: Chi-squared; *p*: *p*-value.

Analyzing Table 2, it was found that, of the 79 individuals who had consumed illicit substances, 36 (45.5%) had been consuming for 10 years or less, and 31 (39.2%) were in the 37–44 age group. There was a statistically significant relationship between years of consumption and age group (χ^2^(4) = 8.26; *p* = 0.015).

### Relationship between the use of illicit substances and attendance at the rehabilitation program and the most prevalent oral manifestations

We didn´t find statistically significant relationships between the different oral manifestations and consumption of illicit substances as shown in Table 3.

**Table 3:** Oral manifestations and substance use and rehabilitation program.

		Use of illicit substances		χ^2^	*p*	Rehabilitation program		χ^2^	*p*
		Yes	No			Yes	No		
		*n* (%)	*n* (%)			*n* (%)	*n* (%)		
Caries lesion	Yes	43 (86.0)	7 (14.0)	0.917	0.338	19 (42.2)	26 (57.8)	0.390	0.532
	No	27 (93.1)	2 (6.9)			12 (35.3)	22 (64.7)		
Chewing difficulty	Yes	30 (85.7)	5 (5.1)	0.521	0.470	20 (52.6)	18 (47.4)	5.51	0.019
	No	40 (90.9)	4 (9.1)			11 (26.8)	30 (73.2)		
Tooth sensivity	Yes	35 (89.7)	4 (10.3)	0.914	0.339	16 (42.1)	22 (57.9)	0.252	0.616
	No	35 (87.5)	5 (12.5)			15 (36.6)	26 (63.4)		
Oral mucosa inflammation	Yes	31 (88.6)	4 (11.4)	0.098	0.754	12 (35.3)	22 (64.7)	0.390	0.532
	No	39 (88.6)	5 (15.1)			19 (42.2)	26 (57.8)		
Periodontitis	Yes	33 (91.7)	3 (8.3)	0.603	0.431	18 (51.4)	17 (48.6)	3.92	0.048
	No	37 (86.0)	96(14.0)			13 (29.5)	31 (70.5)		
TMD	Yes	22 (88.0)	3 (12.0)	0.013	0.908	8 (30.8)	18 (69.2)	1.17	0.280
	No	48 (88.9)	6 (11.1)			23 (43.4)	30 (56.6)		
Bruxism	Yes	23 (92.0)	2 (8.0)	0.417	0.518	7 (28.0)	18 (72.0)	1.94	0.164
	No	47 (87.0)	7 (13.0)			24 (44.4)	30 (55.6)		
Xerostomia	Yes	22 (95.7)	1 (4.3)	1.60	0.207	13 (52.0)	12 (48.0)	2.50	0.114
	No	48 (85.7)	8 (14.3)			18 (33.3)	36 (66.7)		
Gingivitis	Yes	21 (87.5)	3 (12.5)	0.042	0.838	4 (17.4)	19 (82.6)	6.50	0.011
	No	49 (89.1)	6 (10.9)			27 (48.2)	29 (51.8)		
Rampant caries lesion	Yes	14 (93.3)	1 (6.7)	0.410	0.522	9 (56.3)	7 (43.8)	2.44	0.119
	No	56 (87.5)	8 (12.5)			22 (34.9)	41 (65.1)		
Gingival bleeding	Yes	14 (82.4)	3 (17.6)	0.839	0.360	6 (37.5)	10 (62.5)	0.025	0.873
	No	56 (90.3)	6 (9.7)			25 (39.7)	38 (60.3)		

*n*: number; χ^2^: chi square; *p*: *p*-value; TMD: temporomandibular disorders.

When we relate the oral manifestations to whether they attended the rehabilitation program, we can see that of the 38 participants who had difficulty chewing, 20 (52.6%) attended the rehabilitation program, while of the 41 who did not have difficulty chewing, only 11 (26.8%) attended the same program (Table 3). There was therefore a statistically significant relationship between attending the rehabilitation program and chewing difficulties (χ^2^(1) = 5.51; *p* = 0.019). The same was true for periodontitis (χ^2^(1) = 3.92; *p* = 0.048), where out of a total of 35 individuals who had it, 51.4% were attending the methadone program. Regarding gingivitis (χ^2^(1) = 6.50; *p* = 0.011), although a statistically significant difference was also found, it was the opposite. That is, out of a total of 23 inmates with gingivitis, 19 (82.6%) were not attending the methadone program. Regarding the oral manifestations that did not show statistically significant differences, the majority (57.8%) of those who had caries lesions were not attending the rehabilitation program.

The same was true for participants who had tooth sensitivity (57.9%), inflammation of the oral mucosa (64.7%), TMD (69.2%), bruxism (72.0%) and bleeding gums (62.5%). Although xerostomia and rampant caries lesions did not show statistically significant differences, it was observed that these manifestations occurred in a higher percentage (52.0% and 56.3%, respectively) of inmates who attended the methadone program.

### Relationship between the most prevalent oral manifestations and the type of illicit substances consumed

When we related the type of oral manifestations to the type of illicit substances consumed, specifically cannabis or hard drugs (cocaine, heroin), we found that the highest prevalence of oral manifestations was observed in prisoners who had consumed ‘hard’ illicit substances than cannabis (Table 4). We found a statistically significant relationship between difficulty chewing and the type of substance consumed (χ^2^(1) = 5.06; *p* = 0.024), and of the 30 individuals who showed this lesion, 26 (86.7%) consumed hard drugs. Of the 14 individuals who had a rampant caries lesion, all consumed hard drugs, and this relationship was statistically significant (χ^2^(1) = 6.52; *p* = 0.011).

**Table 4:** Relationship between the most prevalent oral manifestations and the consumption of cannabis and hard drugs.

		Type of illicit substances		χ^2^	*p*
		Cannabis	Hard drugs		
		*n* (%)	*n* (%)		
Caries lesion	Yes	10 (23.3)	33 (76.7)	0.852	0.356
	No	9 (33.3)	18 (66.7)		
Chewing difficulty	Yes	4 (13.3)	26 (86.7)	5.06	0.024
	No	15 (37.5)	25 (62.5)		
Tooth sensivity	Yes	8 (22.9)	27 (77.1)	0.650	0.420
	No	11 (31.4)	24 (68.6)		
Oral mucosa inflammation	Yes	5 (16.1)	26 (83.9)	3.41	0.065
	No	14 (35.9)	25 (64.1)		
Periodontitis	Yes	6 (18.2)	27 (81.8)	2.54	0.111
	No	13 (35.1)	24 (64.9)		
TMD	Yes	3 (13.6)	19 (86.4)	2.96	0.085
	No	16 (33.3)	32 (66.7)		
Bruxism	Yes	7 (30.4)	16 (69.6)	0.188	0.665
	No	12 (25.5)	35 (74.5)		
Xerostomia	Yes	5 (22.7)	17 (77.3)	0.316	0.574
	No	14 (29.2)	34 (70.8)		
Gingivitis	Yes	7 (33.3)	14 (66.7)	0.581	0.446
	No	12 (24.5)	37 (75.5)		
Rampant caries lesion	Yes	0 (0.0)	14 (100.0)	6.52	0.011
	No	19 (33.9)	37 (66.1)		
Gingival bleeding	Yes	2 (14.3)	12 (85.7)	1.46	0.226
	No	17 (30.4)	39 (69.6)		

*n*: number; χ^2^: chi square; *p*: *p*-value.

## Discussion

This study included 91 inmates from a prison in northern Portugal. The number of participants, although with variations, is in line with international studies, namely those studies carried out in Spain (*n* = 63) [15], Brazil (*n* = 112) [5], Israel (*n* = 69) [16] and Finland (*n* = 89) [17]. It is worth highlighting that a study carried out in Portugal, also in a prison setting, which evaluated addiction treatment had a total of 78 participants [18]. Like the studies carried out by Raija Vainionpää [17] and Chaturvedi et al. [19], most of the participants in our study belonged to the 36–44 age group, with an average age of 41. As found in this study, which showed that most participants had basic education (2nd and 3rd cycles), the literature mentions that a lower level of education among prisoners is an increased risk factor for the consumption of illicit substances [1]. Our results are corroborated by several studies, namely those by Caravaca-Sanchez et al. [1] and Al Bush et al. [6], where lower levels of education predominate.

Regarding smoking habits, this study showed that many inmates were smokers (89.0%) and that almost half of them (44.0%) smoked a large amount every day, ranging from 11 to 20 cigarettes. Smoking is known to be an important risk factor for the appearance of oral manifestations, and its risk increases when it is combined with other risky behaviors, such as the consumption of illicit substances [20]. In this sense, and as might be expected, our study showed that the relationship between smoking and the consumption of illicit substances was statistically significant. In common sense, most of the time, smoking habits are the trigger for the discovery of other substances [21].

There are several studies that corroborate our results, reporting a high prevalence of smoking. Priwe et al. [22] and Vainionpää [17] report a smoking prevalence of 80% or more. One of the reasons reported by inmates regarding the high percentage of smokers and the large amount of tobacco consumed each day is the stress they suffer, saying that they smoke the most at night because they are locked in their cells and don’t have any kind of abstraction.

Another risky behavior normally associated with smoking, as well as with the consumption of illicit substances, is alcohol consumption [21, 23], but the latter was not verified in our study. The respondents who reported drinking alcoholic beverages only mentioned doing so in the context of precarious exits from Prison Establishment (PE). One of the possible reasons for obtaining such a low prevalence of alcohol consumption is that the question was asked in the ‘present’ tense, and inmates often justified their answer with ‘there isn’t any in here’. The literature essentially shows a prevalence of alcohol consumption habits prior to entering prison [17, 24], but there are other studies that show alcohol consumption during imprisonment [25].

Meanwhile, the consumption of illicit substances showed a high prevalence, with 86.8% reporting having used one or more of them. Similar to our study, several international studies have reported significant rates of illicit substance use, which may vary depending on whether the use was before or during imprisonment. This is because the data on consumption during the period of imprisonment is scarce in national and international literature, due to the difficulty of asking prisoners to report their actual consumption in a context where it is prohibited [24]. As an example, a study carried out in France in 2019 by Rousselet et al. [24] reports a prevalence of daily consumption before incarceration of 58.9%, decreasing to 31.1% during imprisonment. Also, during the period of imprisonment, a study conducted in Mexico found a prevalence of 53.8% illicit substance use [26], similar to a study carried out in Spain, where the prevalence was 59.9% [1]. In a study conducted in Portugal, which mentions the European Monitoring Center for Drugs and Drug Addiction, the prevalence of illicit substance use was reported to in the range of 1 to 50%, with a considerable percentage claiming to consume them regularly [27]. With respect to higher consumption patterns, a study in London reported a prevalence of 79% of illicit substance use before being sent to prison [5], and in England, it was 70% in the 12 months prior to imprisonment [28].

The most reported illicit substances consumed by prisoners were cannabis, heroin and cocaine, which together accounted for 29.1%.

In line with this, a study carried out in France [24] found that prior to imprisonment, cannabis was the most consumed illicit substance (49%), much more prevalent than cocaine (16%) and heroin (8.8%). Also, in a study carried out in England by Ramsay et al. [28], in the 12 months prior to imprisonment, cannabis was the most widely consumed illicit substance with a prevalence of 55%, followed by heroin (35%) and cocaine (28%). In our study, the prevalence rates were considerably lower for the latter two: 3.8% and 5.1%, respectively.

Similarly, although with lower rates, in our study, cannabis alone had a prevalence of consumption of 20.3%. The literature points out that one of the reasons why this substance has a high rate of consumption may be related to the fact that it reduces anxiety in relation to the consumption of other illicit substances [15].

At the other extreme, with only 1.3%, there was consumption of the combination of ‘cocaine, amphetamines and ecstasy’, with no participant mentioning consumption of only amphetamines or ecstasy. This finding was in contrast to some other studies which indicate a considerable prevalence of their use [29].

Although there are studies which conclude that substance abuse is not limited to a specific age group [29], a statistically significant relationship could be seen between the consumption of illicit substances and the age group of the participants. In this sense, it was found that most individuals who consumed illicit substances (45.5%) consumed them 10 years ago or less, and 39.2% were in the 37–44 age group. In this group, there were eight inmates who started using illicit substances more than 20 years ago, which suggests that they started using them earlier, compared to those in the >44 age group, for example, who would be expected to have been using for longer. It should be noted that even within the 25–36 years age group, there are 48.1% of inmates who started using illicit drugs 11–20 years ago, which indicates that some had started using them in their pre-adolescence. This data is in line with the findings of Ramsay et al. [28].

As Saraiva [18] points out, Agra’s 1997 pioneering work in Portugal, which characterized the ‘drug’–crime relationship, or rather, the relationship that deviant individuals have with psychoactive drug consumption, already established that contact with the so-called ‘soft’ drugs begins very early and can start as early as the age of 11, varying up to the age of 22 for the consumption of these drugs.

When we collected our data, many of the inmates revealed that their use of illicit substances began very early in their lives, starting with cannabis. Perhaps this is also the reason why this substance had such a high rate of consumption. One study analyzed the onset of cannabis use, in which the average age of onset was 14.4 years [15]. The research by Ramsay et al. [28] goes even further, reporting that the use of so-called recreational drugs such as cannabis and ecstasy is significantly more common among the younger age group (15 to 20 years), while the use of so-called destructive substances such as cocaine and heroin is more common in the group aged between 21 and 29. The rate of amphetamine consumption was basically the same for these two age groups.

### Prevalence of oral manifestations

According to the literature, in addition to an increase in oral diseases among illicit substance users compared to non-users [9, 23], there seems to be a consensus profile of oral manifestations, in which caries lesions, periodontal diseases, xerostomia, bruxism and TMD predominate. Oral mucosa lesions can also be found in this range of consumers, although in our study it was one of the least observed manifestations [23, 29].

However, the fact that polyconsumption predominated in our sample for most illicit substances makes it difficult to indicate an association between their use and the oral diseases they cause, also due to the frequent association with tobacco and alcohol [29].

Caries lesion, the most prevalent oral manifestation in this study at 61.0%, is one of the manifestations most reported in other studies and is widely associated with the consumption of various types of illicit substances [6, 9, 11]. Rampant caries lesions were also observed in some individuals (*n* = 15), and the literature shows that opioid consumption, and more specifically heroin, can cause such conditions [30]. In our study, heroin does not have a high consumption rate on its own, but it is consumed together with other substances. The difficulty in chewing, attributed essentially by the inmates to missing teeth, was also confirmed by our study, where most participants had one or more missing teeth. However, the reasons that led to the loss of teeth were not collected, which limits the reasoning behind this manifestation. However, there are studies that tell us that the consumption of illicit substances may be the reason for tooth loss [17, 22, 23].

Another oral manifestation that was widely reported, with a prevalence of 48.8%, was tooth sensitivity. Although most existing studies point to this oral symptom as essentially an effect of the consumption of synthetic substances such as ecstasy and methamphetamines [30], it has also been reported because of the use of stimulants such as cocaine, which in this study seems to be the most logical association [31].

At 42.7%, there was inflammation of the oral mucosa, which was also found in a considerable number of participants. It was closely related to gingival bleeding, but this was not reported to the same extent. This difference may be because the variable ‘bleeding gums’ was reported by the inmate himself, unlike ‘inflammation of the oral mucosa’ which was assessed through clinical observation.

The prevalence of periodontal disease was also notable, with 36 inmates (43.9%) showing periodontitis and 23 (28.0%) reporting gingivitis. There is a consensus in the literature that periodontal disease is frequently observed in users of illicit substances [8, 9]. Smoking and the decrease in salivary flow induced by illicit substances are two important risk factors [32].

Oral manifestations such as bruxism and TMD are commonly associated with the consumption of illicit substances [33]. The former, with 30.5% of observations, corroborates that it is a prevalent manifestation in illicit substance users, one of the consequences of which is dental attrition, which was also detected in our study [34]. This study also revealed a moderate prevalence (32.9%) of self-reported TMD symptoms, in line with a study of Finnish inmates [17], where it reached 33.0%, and is also in line with the research by Pantoja et al. [35]. This prevalence, as was the case in our study, is based on clinical diagnoses only, and there is no possibility of imaging tests in the prison environment.

There is a consensus in the literature regarding xerostomia, stating that a wide range of illicit substances have an impact on salivary flow and, consequently, increase the vulnerability to dental caries lesions [36]. Opiates (e.g. heroin and methadone) and cannabis are known to cause hyposalivation [10]. In this sense, our study confirms this data, with a prevalence of 29.3%.

There are also many case reports on methamphetamine users describing the so-called meth mouth [37]. In our research, as in other epidemiological studies which report that their dentition is, in many cases, much less seriously affected than the one portrayed, we can only associate rampant caries lesions with this profile, which can lead to partial or total edentulism. There is also xerostomia, periodontal disease and bruxism [3, 37].

There are also 31 (34.1%) inmates on the methadone program, which can trigger oral repercussions [2]. Of the oral manifestations most reported because of taking methadone, xerostomia, caries lesions and periodontal disease are the most prevalent [2, 22, 38]. In this study, periodontitis and difficulty chewing were the most statistically significant oral manifestations. Regarding the latter, opioids in general can cause tooth loss [31, 39] and, since methadone is considered an opioid, this could also be one of the reasons for the high rates of tooth loss and, consequently, chewing difficulties.

Finally, it was found that regardless of the type of oral manifestation observed, the highest prevalence occurred in individuals who had been using illicit substances for 10 years or less, which is in line with the years of consumption which, as mentioned above, was the most prominent time. Once again, ‘difficulty chewing’ had a statistically significant relationship and, as has already been reported, this can be attributed to the consumption of a wide variety of illicit substances. The longer the exposure to these substances, the greater the dysfunction of the stomatognathic system, namely chewing difficulties [29]. The other oral manifestation that was statistically significant was bruxism, which can be largely associated with the use of stimulant drugs such as cocaine, which in our study had a significant prevalence of consumption [37].

### Limitations of the study and future research prospects

Some limitations are present in this study such as some subjectivity of questions inherent to this kind of study. Without ever forgetting that the inmates’ privacy would also have to be safeguarded, the way we found to combat this possible bias was to ask the questions in the past tense.

As regards recommendations for future research, it would be interesting if, in addition to the risk factors for the consumption of illicit substances studied by us, more data were collected, such as a series of prison variables like whether or not the length of the sentence had an influence on the rates of consumption of illicit substances, a comparison between genders and an analysis of other sociodemographic factors like social class, and also the collection of age at the time of imprisonment in order to obtain the exact ages at which consumption began in a more detailed and individualized way. With this, it would be possible to draw up a more complete profile to take more preventive and/or urgent action. It would also be interesting to extend the national sample to several PE to assess possible differences in consumption patterns and, consequently, differences in the oral manifestations observed. In this sense, our study could also lead to new discoveries on how PE are prepared to provide adequate treatment for consumers, whether it be rehabilitation programs or alerting the dentists who work there to provide inmates with adequate information and guidance to improve their habits and behaviors.

## Conclusion

The oral manifestations resulting from the consumption of illicit substances most commonly observed in this study were caries lesions, chewing difficulties, periodontal diseases, xerostomia, TMD and bruxism, with the most consumed illicit substances being cannabis, cocaine and heroin. It was observed that younger individuals had longer patterns of consumption. Smoking proved to be one of the most common behaviors among the inmate population. There was a tendency to combine this behavior with the consumption of illicit substances.

Considering the scarcity of research on this topic, this study provides new scientific contribution to the field, aiming to alert the need for the implementation of effective strategies for the early diagnosis and treatment of oral manifestations resulting from the use of illicit substances in prison.

## Data Availability

The data can be accessed by contacting the corresponding author.
